# Retention and Transfer of Cognitive Bias Mitigation Interventions: A Systematic Literature Study

**DOI:** 10.3389/fpsyg.2021.629354

**Published:** 2021-08-12

**Authors:** J.E. (Hans) Korteling, Jasmin Y. J. Gerritsma, Alexander Toet

**Affiliations:** Netherlands Organisation for Applied Scientific Research (TNO) Human Factors, Soesterberg, Netherlands

**Keywords:** cognitive biases, bias mitigation, retention, transfer of training, training interventions, neural networks, systematic literature study

## Abstract

Cognitive biases can adversely affect human judgment and decision making and should therefore preferably be mitigated, so that we can achieve our goals as effectively as possible. Hence, numerous bias mitigation interventions have been developed and evaluated. However, to be effective in practical situations beyond laboratory conditions, the bias mitigation effects of these interventions should be retained over time and should transfer across contexts. This systematic review provides an overview of the literature on retention and transfer of bias mitigation interventions. A systematic search yielded 52 studies that were eligible for screening. At the end of the selection process, only 12 peer-reviewed studies remained that adequately studied retention over a period of at least 14 days (all 12 studies) or transfer to different tasks and contexts (one study). Eleven of the relevant studies investigated the effects of bias mitigation training using game- or video-based interventions. These 11 studies showed considerable overlap regarding the biases studied, kinds of interventions, and decision-making domains. Most of them indicated that gaming interventions were effective after the retention interval and that games were more effective than video interventions. The study that investigated transfer of bias mitigation training (next to retention) found indications of transfer across contexts. To be effective in practical circumstances, achieved effects of cognitive training should lead to enduring changes in the decision maker's behavior and should generalize toward other task domains or training contexts. Given the small number of overlapping studies, our main conclusion is that there is currently insufficient evidence that bias mitigation interventions will substantially help people to make better decisions in real life conditions. This is in line with recent theoretical insights about the “hard-wired” neural and evolutionary origin of cognitive biases.

## Introduction

People constantly form judgments and make decisions, both consciously and unconsciously, without certainty about their consequences. The decision to take another job, to start a relationship, or to visit a friend is generally made without knowing beforehand how internal and contextual “success-factors” will develop, or what will happen when these decisions are really carried out. When making these kinds of decisions our thinking is characterized by systematic distortions that often seem to violate the rules of logic and probability. These violations may be manifested in cognitive biases (e.g., Tversky and Kahneman, 1974; Haselton et al., 2005; LeBoeuf and Shafir, 2005; Toet et al., 2016; Korteling and Toet, 2020). Cognitive biases can be generally described as systematic and common tendencies, inclinations, or dispositions that skew or distort decision making processes in ways that may make their outcomes inaccurate or suboptimal (Lichtenstein and Slovic, 1971; Tversky and Kahneman, 1981). Biases occur in virtually the same way in many different decision situations (Shafir and LeBoeuf, 2002; Kahneman, 2011a; Korteling et al., 2018). They distort our thinking in very specific ways, are largely implicit and unconscious, and feel quite natural and self-evident (Risen, 2015; Korteling et al., 2018). That is why they are often termed “intuitive” (Kahneman and Klein, 2009), “irrational” (Shafir and LeBoeuf, 2002), or a-rational (Korteling and Toet, 2020). People tend to detect biased reasoning in others more than in ourselves and we typically feel quite confident about our decisions and judgments, even when we are aware of our cognitive biases and when there is hardly any evidence to support them (Pronin et al., 2002; Risen, 2015; Scopelliti et al., 2015). The robust, pervasive and persistent cognitive bias phenomenon is extensively described and demonstrated in the psychological literature (Kahneman and Tversky, 2000; Hastie and Dawes, 2001; Shafir and LeBoeuf, 2002; Kahneman, 2011a; Korteling et al., 2018). Some well-known biases are: Belief bias (the tendency to base the power or relevance of an idea on the credibility of the conclusion instead of on the argument: Evans et al., 1983), Confirmation bias (the tendency to select, interpret, focus on and remember information in a way that confirms one's preconceptions, views, and expectations: Nickerson, 1998), Fundamental attribution error. (the tendency to overestimate the influence of personality, while underestimating the importance of situational factors when explaining events or behaviors of other people: Jones and Harris, 1967; Gilbert, 1998), Hyperbolic discounting (the tendency to prefer a smaller reward that arrives sooner over a larger reward that arrives later: Alexander and Brown, 2010), Outcome bias (the tendency to evaluate a decision based on its outcome rather than on what factors led to the decision: Baron and Hershey, 1988), Representativeness bias (the tendency to judge the likelihood of an entity by the extent to which it “resembles the typical case” instead of by its simple base rate: Tversky and Kahneman, 1982b), the Sunk-cost fallacy (the tendency to consistently continue a chosen course or investment with negative outcomes rather than alter it: Arkes and Ayton, 1999) or Social proof (the tendency to copy the actions and opinions of others Cialdini, 1984).

Biased thinking can result in outcomes that are quite acceptable in everyday situations, especially when the time and processing cost of reasoning is taken into account (Simon, 1955; Gigerenzer and Gaissmaier, 2011). This is for example the case under time pressure or when relevant or available information is too extensive or detailed, or when no optimal solution is evident (e.g., “Bounded Rationality”; Simon, 1955). In these cases, we often use practical decision routines (“heuristics”), characterized by a high ratio of benefits to cost in terms of the quality of the outcomes relative to invested time, effort, and resources (Gigerenzer and Todd, 1999). However, biased, or heuristic reasoning often leads to outcomes that deviate from what may be considered optimal, advisable, or utile (in relation to our personal objectives). These deviations are also not random, but very specific and systematic: in a wide range of different conditions, people show the same, typical tendencies in the way they pick up and process information to judge and decide. This applies to (almost) everybody, at all levels and in all parts of society, not only in our daily life, but also in professional companies, organizations, and institutions. This may have substantial practical consequences, for example in the context of corporate government or policymaking when decision making is often very complex with far-reaching consequences (Flyvbjerg, 2007; Vis, 2011; Bellé et al., 2018). For example, in policymaking the outcomes of a plan can be “framed” in terms of gains, which leads to a preference of risk avoidance, whereas framing the outcomes in terms of losses can lead to risk seeking (Tversky and Kahneman, 1981; Plous, 1993; Mercer, 2005). This means that people base their decisions on the way a problem is formulated rather than on its content. Besides framing, the number of choice alternatives can influence decision making (Bellé et al., 2018). Decision makers prefer the status quo when the number of alternatives is high (Status quo bias: the tendency to prefer the current state of affairs; Samuelson and Zeckhauser, 1988). These are just a few practical examples of many factors that have been shown to systematically affect the choices people make (Kahneman, 2011a).

Mitigating cognitive biases may lead to better decision making on all levels of society, which could substantially promote long-term human well-being. Substantial research has already been conducted as to whether and how cognitive biases can be mitigated. Merely teaching (abstract) knowledge on the existence and nature of cognitive biases has appeared insufficient to mitigate them (e.g., Fischoff, 1982; Nisbett et al., 1983; Fong et al., 1986; Larrick, 2004; Beaulac and Kenyon, 2018). Therefore, more elaborate training methods and tools for debiasing have been developed, where people are intensively educated and trained how to mitigate one or more cognitive biases (Poos et al., 2017). One method is to ask people to consider why their initial judgments could be wrong. This strategy is called “*consider the opposite*” and has been shown to reduce various biases (Arkes et al., 1988; Mussweiler et al., 2000). Most studies investigate the bias mitigating effect just after finishing the training while using the same type of tasks that were also used during the training (Larrick et al., 1990; Clarkson et al., 2002; Cheng and Wu, 2010). To be truly effective in real life, however, achieved effects of cognitive training should lead to enduring changes in the decision maker's behavior and should generalize toward other task domains or training contexts (Fong and Nisbett, 1991). This means that the *retention of training* should be high, and the bias mitigation effects should last for a longer time than just a brief period immediately after the training. It cannot simply be assumed that an intervention that is effective right after training, will still be effective at a later time (Schmidt and Bjork, 1992). In addition, to have practical value, the bias mitigation effects should transfer to real-life decision situations beyond the specific training environment or context. So, people should be able to apply what they have learnt in a bias mitigation training to more than just one specific type of problem or situation. To date, there is no study that systematically reviews and analyzes the retention and transfer effects of bias mitigation interventions. Therefore, the present systematic literature study provides an overview of the available studies on retention and transfer of bias mitigation interventions.

## Method

### Protocol and Systematic Search

Four databases were used: Scopus, Web of Science, PubMed and Psychinfo. For the search, two elements were of interests: debiasing (title/abstract) and retention or transfer (all fields). These elements were adapted to the respective databases. For example, the search string used in Scopus was: (title-ABS-key [“bias mitigation” OR debiasing] AND ALL [retention OR transfer]). The field specifications were made to search for papers that deal with retention and transfer, within the more general topic of debiasing.

### Inclusion and Exclusion Criteria

Studies where included if they investigated the effectiveness of cognitive bias mitigation interventions. Cognitive biases had to be investigated explicitly, while studies investigating an improvement of performance in a broader sense were excluded (Fong and Nisbett, 1991; Kostopoulou et al., 2017). Secondly, either retention of debiasing or transfer were investigated. For retention, there had to be one measurement of bias at least two weeks after the intervention. Although not very long for practical purposes, this two-week period may be considered sufficient to demonstrate retaining training effects (Fong and Nisbett, 1991). The transfer investigated had to include real behavior transcending the specific training conditions, problem structures, and domains (domain independence). Thus, to demonstrate (true) transfer, subjects should show improved behavior or skills surreptitiously measured beyond the training context, in different and realistic field conditions. Studies measuring “*near transfer*,” for instance to another set of similar test items, situation assessments, or obvious questions that are considered to measure cognitive bias, were excluded. So, asking subjects to assess situations, how they would behave or if they think that they have learned, were not considered as sufficient measures of transfer. Finally, only primary experimental studies were used.

### Data Collection and Analysis

After the systematic search, duplicates were removed, and titles and abstracts were carefully screened to determine if the article addressed cognitive bias mitigation. For the articles meeting this criterium, full texts were obtained to determine if retention and/or “far transfer” of the bias mitigation intervention was indeed assessed. Finally, relevant data were extracted.

## Results

**Figure 1:**
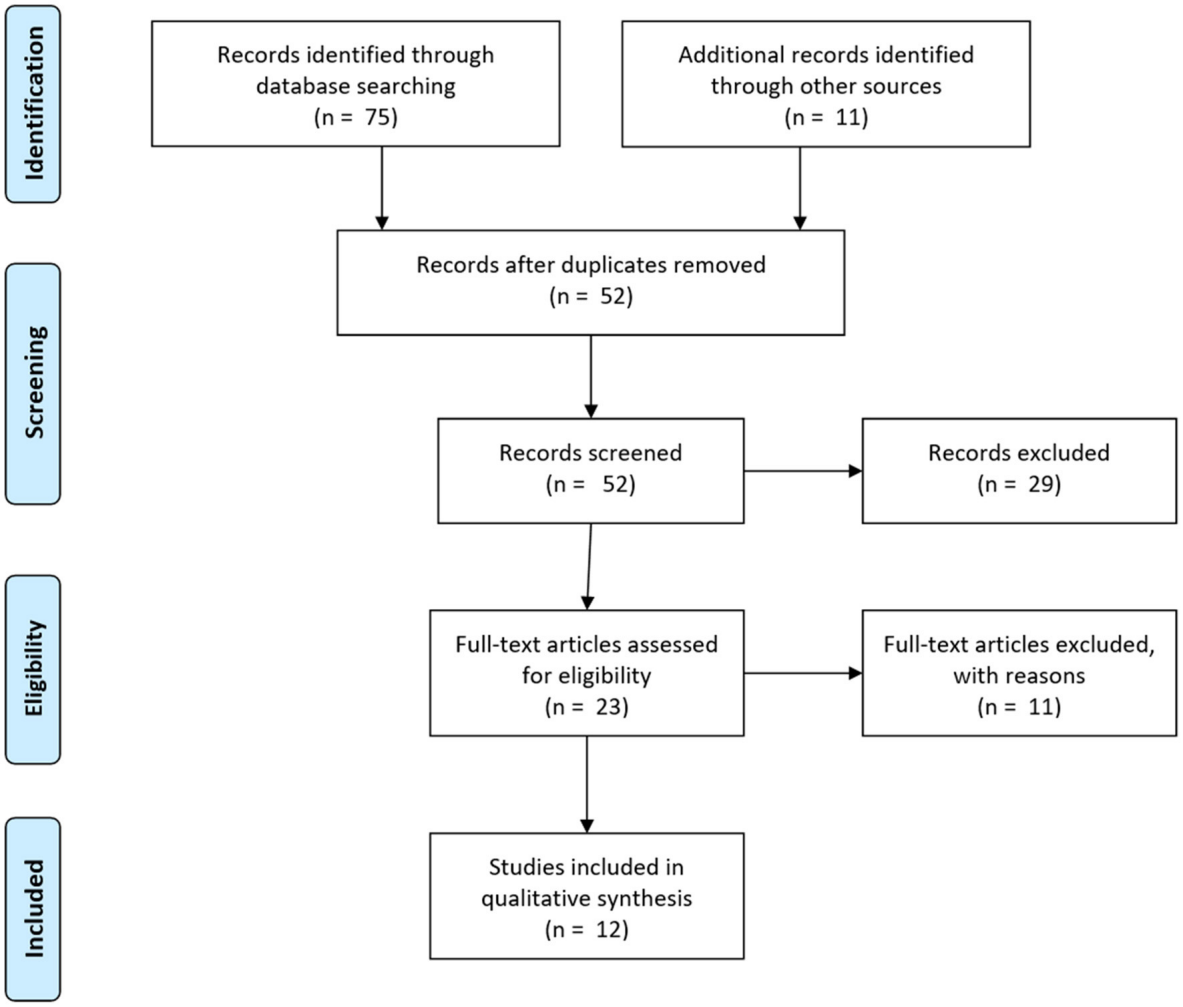
PRISMA 2009 flow diagram of the study selection process (adapted from Moher et al., 2009).

From the 75 articles returned by the initial search and the 11 papers that were identified through other sources, only 12 articles remained after selection. Figure 1 illustrates the selection process. Of the 86 initial articles, 34 were duplicates. Of the remaining 52 articles, 29 did not (properly) investigate cognitive bias mitigation. They rather studied some other form of bias in another field (10 articles), investigated the existence of only a particular cognitive bias (four articles), or were not experimental studies at all (15 articles). Of the 23 subsequently assessed articles, six only assessed “*near transfer,”* which was not the interest of the present study, and five studies did not study the transfer or retention of the effects of interventions. The remaining 12 articles that were included in this study, described 17 single experiments in total, all of which investigated retention, while only one study focused on real transfer. Table 1 lists the definitions of the different cognitive biases and the number of mitigation experiments in which these biases were investigated in these 12 studies.

**Table 1:** Definitions of cognitive biases and number of mitigation experiments in which these biases were studied.

**Type of bias**	**Definition**	**Number**
Anchoring bias	“overweighting the first information primed or considered in subsequent judgment” (Tversky and Kahneman, 1974)	6
Base rate neglect	“the tendency of people to neglect statistical base rate information when making decisions” (Tversky and Kahneman, 1982a; Fong et al., 1986)	1
Bias blind spot	“perceiving oneself to be less biased than one's peers” (Pronin et al., 2002; Scopelliti et al., 2015)	10
Confirmation bias	“gathering and interpreting evidence in a manner confirming rather than disconfirming the hypothesis being tested” (Nickerson, 1998)	10
Fundamental attribution error a.k.a. Correspondence bias	“attributing the behavior of a person to dispositional rather than to situational influences” (Jones and Harris, 1967; Gilbert, 1998) “tendency to make correspondent inferences” or “neglect of external demands” (Scopelliti et al., 2018),	10
Covariation detection	“how people judge whether a component has an effect, with or without taking into account the other elements of the contingency table” (Stanovich and West, 1998)	1
Framing effect	“the tendency of people to decide differently when the same information is worded differently” (Tversky and Kahneman, 1981; Plous, 1993)	1
Insensitivity to sample size	“people's tendency to disregard the fact that small samples don't follow the laws of big samples” (Tversky and Kahneman, 1974)	1
Outcome bias	“the tendency of people to evaluate quality of decisions based on their outcome” (Baron and Hershey, 1988)	1
Overconfidence bias	“the tendency of people to perceive their ability as better than it actually is” (Aczel et al., 2015)	1
Projection bias	“assuming others' emotions, thoughts, and values are similar to one's own” (Epley et al., 2004; Robbins and Krueger, 2005)	4
Regression to the mean	“the tendency of people not to take into account that after an extreme value the next value will more probably be closer to the mean” (Bazerman, 2005)	1
Representativeness bias	“using the similarity of an outcome to a prototypical outcome to judge its probability” (Kahneman and Tversky, 1972)	5
Sunk cost fallacy	“people's tendency to continue an activity if they have already invested money, time or effort in it” (Arkes and Blumer, 1985; Arkes and Ayton, 1999)	1

Below we will summarize the 12 studies that resulted from our selection. These studies are discussed in chronological order, with the publication dates of the interventions as the first level and the year of the study as the second level order parameter.

The article on retention by Dunbar et al. (2014) investigated the effectiveness of the MACBETH debiasing game (Dunbar et al., 2013) played either once or twice for 30 or 60 min and with or without explicit feedback on biases, and a control condition in which a bias training video was presented. The game and video both aimed to mitigate the fundamental attribution error and the confirmation bias. The results showed that all conditions mitigated the biases to some extent, and this reduction was retained over a period of 8 weeks. The authors present no conclusive evidence that any of the conditions tested consistently and significantly outperforms the others. The results suggest that repeated playing of the game with explicit bias feedback most effectively enhanced the mitigation of the confirmation bias. However, this result was confounded by a marginally significant 3-way interaction among training type, duration, and the different locations at which the tests were performed.

The article on retention by Bessarabova et al. (2016) investigated the effectiveness of the MACBETH debiasing game played either once or twice for 30 or 60 min, either without (implicit condition) or with (hybrid condition) explicit feedback on biases and a control condition in which a bias training video was presented. The game and video both aimed to mitigate the bias blind spot. Their first experiment showed that playing the game twice reduced the bias blind spot right after the intervention significantly more than playing it only once, but both conditions yielded a similar reduction after 8 weeks. They found no effect of feedback type (hybrid or implicit) and play duration. Their second experiment showed similar results: again, there was no effect of feedback type (just-in-time vs. delayed) and play duration. Playing the game twice reduced the bias blind spot right after the intervention significantly more than playing it once, but both conditions again resulted in a similar reduction after 8 weeks. Their third experiment showed no difference between playing the game in a single- or multiplayer mode, and again no effect of repetition (playing only once or twice) or duration. Overall, this study found that gameplay reduced bias blind spot from pretest to 8-week posttest.

The article on retention by Dunbar et al. (2017) investigated the effectiveness of the MACBETH debiasing game (Dunbar et al., 2013) played either once or twice for 30 or 60 min in a single- or multi-player mode with either immediate or delayed feedback on biases, and a control condition in which a bias training video was presented. The game and video both aimed to mitigate the fundamental attribution error and the confirmation bias. Their first experiment showed that playing the debiasing game in a single-player mode significantly reduced both investigated biases right after the intervention, and this reduction was retained over an 8-week period (the bias scores immediately after the intervention and after 8 weeks were not significantly different). Game play outperformed the training video in all conditions. The training video did not mitigate the confirmation bias. For both biases there was no effect of game play duration and feedback mode (immediate or delayed). Repeated playing of the game only enhanced the mitigation of the confirmation bias but did not mitigate the fundamental attribution error. Their second experiment replicated some of the results from the first one, showing that longer duration and repeated play were more effective in mitigating confirmation bias than shorter duration and the single-play game, but were not more effective in mitigating the fundamental attribution error. Game playing was overall more effective in reducing both biases than the training video. Playing the game in the single-player mode yielded greater confirmation bias mitigation relative to the multi-player version. Overall, the game outperformed the training video in reducing confirmation bias, and even more so when repeatedly played for longer durations.

The article on retention by Clegg et al. (2014) investigated the effectiveness of a (Flash-based) debiasing game played either once or twice spaced by 7–10 days, and a control condition in which a professionally developed training video was used. The game and video both aimed to mitigate the fundamental attribution error, confirmation bias and bias blind spot (see Table 1 for definitions of these biases). The debiasing game significantly reduced all biases investigated (fundamental attribution error, confirmation bias, bias blind spot) right after the intervention and after 8 weeks. The training video had no effect on the confirmation bias, but significantly reduced the fundamental attribution error right after the intervention and after 8 weeks, and the bias blind spot bias after 8 weeks but not right after the intervention. The authors suggest that this surprising (but small) retention effect could arise because watching the video might make participants believe that they were still biased. Playing the game twice only significantly enhanced the retention of the confirmation bias mitigation after 8 weeks, but not immediately after the intervention and had no effect on the other biases. Overall, playing the video game reduced the fundamental attribution error, confirmation bias and bias blind spot and outperformed the video, both immediately after the intervention and at the retention test. Repetition of the training game only affected retention of the confirmation bias mitigation.

The article on retention by Clegg et al. (2015) investigated the effectiveness of a (Flash-based) debiasing game played either once or twice spaced by 10 or 12 days, and a control condition in which a professionally constructed training video was used. The game and video aimed to mitigate the anchoring-, projection- and representativeness biases (see Table 1 for definitions of these biases). The findings regarding anchoring bias are not reliable, due to the unacceptable internal consistency of the questionnaire used (George and Mallery, 2003). For both the projection and representativeness bias, all conditions showed a reduction of bias right after the intervention. After 12 weeks, only gaming still effectively mitigated the projection bias, while both gaming and video watching remained effective for the representativeness bias. Playing the game twice was always superior to watching the video but did not outperform single practice on the immediate posttest. So, the value of repeated practice to the retention of training was only observed after 12 weeks. Therefore, playing the game twice appeared the best method to mitigate projection bias on the long term. This result underlines the importance of testing retention of bias mitigation interventions. The effect sizes in this study were very large (see Supplementary Material), in fact, larger than for the interventions tested in the other studies.

Replicating previous research (Clegg et al., 2014), the article on retention by Shaw et al. (2018) investigated the effectiveness of a (Flash-based) debiasing game played with a character that was either assigned or customized, and a control condition in which a professionally produced bias training video was presented. The game and video both aimed to mitigate the fundamental attribution error, confirmation bias and bias blind spot. The game mitigated the fundamental attribution error and the confirmation bias, both immediately after the intervention and after 8 weeks. Avatar customization had no significant effect on learning outcomes. The game outperformed the training video only for confirmation bias. The training video slightly increased confirmation bias and bias blind spot (but not significantly) in both immediate and 8-week posttests. In contrast to earlier findings (Clegg et al., 2014), none of the interventions reduced the bias blind spot.

The article on retention by Veinott et al. (2013) investigated the effectiveness of the *Heuristica* debiasing game (Mullinix et al., 2013) and a control condition in which a bias training video was presented. The game and training video both aimed to mitigate the confirmation bias, fundamental attribution error and bias blind spot. Their results showed that game playing consistently mitigated all three biases and outperformed the video presentation, and this reduction was retained over a period of 8 weeks. Repeated playing or 3rd person perceptive did not further improve bias mitigation.

The article on retention by Morewedge et al. (2015) also describes and discusses two experiments. The first experiment evaluated the effectiveness of a game (“*Missing*”) compared to that of a related video in mitigating the bias blind spot, confirmation bias and fundamental attribution error (or correspondence bias). While both the game and the video reduced all biases, the game reduced the biases more, both right after the game and eight weeks later. The second experiment evaluated a different game than Experiment 1 and compared it to a related video. The targeted biases were anchoring, representativeness, and social projection. Like in the study of Clegg et al. (2015), the internal consistency of the anchoring bias questionnaire was unacceptable, making the results for this bias meaningless. Both the game and the video reduced biases right after the intervention and this reduction was retained after 12 weeks. The game was more effective in reducing representativeness and social projection right after intervention; 12 weeks later it was only more effective in reducing social projection. These results suggest that serious games can indeed mitigate biases.

The article on retention by Sellier et al. (2019) investigated whether the debiasing effects of the game (“*Missing*”) that was used in the first of the two experiments reported by Morewedge et al. (2015) transfers to fields settings. The game incorporated four debiasing strategies: warning about bias, teaching its directionality, providing feedback, and extensive coaching and training. The targeted bias was the confirmation bias. Measurements of bias blind spot and correspondence bias served as manipulation checks of the efficacy of the training. Replicating previous research (Morewedge et al., 2015), trained participants exhibited significantly lower bias levels on both manipulation checks than untrained controls. Transfer was measured solving a surreptitiously presented business case modeled on the decision to launch the Space Shuttle Challenger. The debiasing effect of the game appeared to transfer to this unrelated problem and was retained up to 52 days. Trained participants were 19% less likely to choose an inferior hypothesis-confirming case solution than untrained ones. This reduction in confirmation bias appeared to result from a reduction in confirmatory hypothesis testing. It must be noted that the definition of the broad “confirmation bias” was restricted to “confirming hypothesis testing.” This may result in a training outcome reflecting more of a specific and limited learned “trick,” rule, or standard operation to disconfirming hypothesis testing when confronted with a hypothesis to be tested. Learning this trick probably will not result in a mitigation of the more general and pervasive thinking tendency to search for, interpret, favor, and recall information in a way that confirms or supports one's prior beliefs (i.e., the confirmation bias is often called “the mother of all biases”: Nickerson, 1998). It is therefore not clear whether the effectiveness of the intervention resulted from this narrow operationalization of the confirmation bias or, in contrast, from the engaging nature of the intervention, the intensive practice and feedback, or the breadth of the training that included practicing the mitigating strategies to different paradigms and domains.

The article on retention by Rhodes et al. (2017) discusses two experiments in which the investigated biases (anchoring bias, projection bias, representativeness bias, bias blind spot, and the confirmation bias), were analyzed as a sum score instead of individually. Since the biases are so diverse in nature, one could question whether the use of a sum score is valid. Presumably, this was done to increase statistical power, which was probably necessary since seven conditions were compared in Study 1 and five in Study 2. Based on the acceptable internal consistency, the questionnaire does seemed reliable. The first experiment evaluated five games (“*MACBETH,” “Heuristica,” “Cycles,” “Missing,” “Enemy of Reason”*), in comparison to a related video that taught the same cognitive biases and an unrelated video that did not teach any biases. All five games and the related video mitigated the biases right after the intervention, while three games outperformed the related video. After eight weeks the overall mitigation effects remained, while now four video games outperformed the related video. The second experiment evaluated the three games that outperformed the video right after the intervention. Two of these outperformed the related video right after the intervention; however, after 12 weeks no game produced better debiasing results than the related video anymore. The related video outperformed the games in teaching the definition of biases, underlining once again that knowledge of biases is not sufficient to achieve substantial bias mitigation in people. The unrelated video was added to investigate whether there was an effect of mere practice, irrespective of its kind. This effect was indeed observed: after eight weeks participants were less biased than right after watching the unrelated video. Therefore, the mere effect of practice seemed to have a “debiasing” effect. However, this effect was smaller than the debiasing effect of the games. To achieve bias mitigation, games appear more effective than videos.

The article on retention by Lee et al. (2016) investigated the effectiveness of a debiasing game and a control condition in which a bias training slideshow lecture was presented. The game and slideshow both aimed to mitigate the anchoring bias and representativeness bias. The combined slideshow plus game condition significantly reduced both the anchoring bias and three types of representativeness bias (stereotype, base rate and sample size, but not the gambler's fallacy) immediately after training. Individually, the game and slideshow had no effect on the anchoring bias. The game and slideshow equally mitigated three of the four (except the gambler's fallacy) tested representativeness biases. However, the effect of the slideshow was significantly reduced after 4 weeks. An immediate posttest showed no significant difference between the three conditions for the representativeness bias. Only the mitigation effects of the combined slideshow plus game condition remained after four weeks, while the effects of the slideshow only and the digital game only conditions were significantly diminished. Overall, the digital game was not more effective than a slideshow lecture as a stand-alone training tool. Combining the slideshow with the digital game led to the most effective bias mitigation, which did not decay after four weeks.

The article on retention by Aczel et al. (2015) evaluated retention of two different training interventions: bias-awareness training, which taught the definitions of the biases and different mitigation strategies, and analogical-debiasing training, which encouraged participants to recognize analogies between different situations in which biases occur. The study also posed one question to the participants asking if they remember having decided differently due to the training. According to our definition of transfer (showing improved behavior beyond the training context, i.e., in different problems or situations), this single subjective question cannot be considered a valid measurement of transfer. The study had a within-subjects design (see Supplementary Material for details). Four weeks after training the only finding regarding retention was that analogical training improved decision-making in regard to the “statistical biases,” a sum score of four biases from the first group of biases (insensitivity to sample size, base rate neglect, regression to the mean, and covariation detection). For individual biases, no significant retention effects were found. These results on retention suggest that training can, although only slightly, mitigate some biases. Transfer was measured by asking participants four weeks after the intervention whether they remembered deciding differently because of the training. This subjective assessment of transfer differs from the more objective measurements of other studies and is probably more sensitive to the disadvantages of self-report measures. Of the participants who received analogical training for the statistical biases, 58% reported “*yes*,” while this was only the case for 32% of the participants who received awareness training for these biases (a significant difference, *p* < 0.01). This may imply that the training affected transfer to some extent. That is, the statistical biases were mitigated the most from the (analogical) debiasing training. Yet, for the other biases, improvement after either the awareness or the analogical training was not found.

A summary of all 12 studies reviewed in this paper is presented in Table 2. See Supplementary Material for a more detailed overview of these studies.

**Table 2:** Summary of the most relevant details of the 12 studies reviewed in this paper.

				**Interventions**				**Biases**							**Effectiveness**	
**References**	**Nr experiments (Nr. Participants)**	**Retention interval (weeks)**	**Transfer**	**Game (Single or Repeated play)**	**Video**	**Slides**	**Training**	**Anchoring bias**	**Bias blind spot**	**Confirmation bias**	**Fundamental attribution error**	**Projection bias**	**Representativeness bias**	**Other**	**Game vs video**	**Single vs repated gameplay**
Dunbar et al. (2014)	1(753)	8		S, R	X					1	1				G ≈ V	R > S
Bessarabova et al. (2016)	3(703, 620, 626)	8		S, R					3						G+	R ≥ S
Dunbar et al. (2017)	2(411, 436)	8		S, R	X					2	2				G > V	R > S
Clegg et al. (2014)	1(390)	8		S, R	X				1	1	1				G ≥ V	R>S
Clegg et al. (2015)	1(191)	12		S, R	X			1				1	1		G > V	R>S
Shaw et al. (2018)	1(234)	8		S	X				1	1	1				G ≥ V	
Veinott et al. (2013)	1(157)	8		S, R	X				1	1	1				G ≥ V	R=S
Morewedge et al. (2015)	2(196, 192)	8, 12		S	X			1	1	1	1	1	1		G > V	
Sellier et al. (2019)	1(290)	1–7	X	S					1	1	1				G+	
Rhodes et al. (2017)	2(301,325)	8, 12		S	X			2	2	2	2	2	2		G ≥ V	
Lee et al. (2016)	1(301)	4		S		X		1					1		G±	
Aczel et al. (2015)	1(154)	4					X	1						8		
Total	17		1	11	8	1	1	6	10	10	10	4	5	8		
**Symbol**	**Meaning**															
=	equally effective as															
≈	comparable effectiveness to															
≥	more effective than or comparable to															
>	more effective than															
+	significant effect															
±	small significant effect															
R	repeated play															
S	single play															

*Listed are the number of experiments reported in each study (with the number of participants included in each experiment), the retention interval (in weeks), whether transfer was investigated, the type of intervention(s) used, the bias(es) investigated, and the relative effectiveness (game vs. video and single vs. repeated gameplay)*.

## Discussion

Bias mitigation training has only practical value when the bias mitigation effects of the intervention are retained over time and transfer across contexts. To explore the evidence for the effectivity of bias mitigation interventions, we performed a systematic literature review on the retention and transfer of bias mitigation interventions. More specifically, this review investigated the (amount and quality of) evidence that bias mitigating interventions yield stable effects that transfer to situations beyond the mere training context. Our main conclusion is that the scientific evidence for both retention and transfer of bias mitigating interventions is scarce. While our literature search initially yielded 52 articles after duplicates were removed, only 12 articles (including 15 experiments on retention and three on retention and transfer) remained after careful selection on relevance and methodological considerations. In addition, all studies (except the one by Aczel et al., 2015) showed considerable overlap (including replications) concerning the biases studied, type of interventions, and decision making domains. The selection criterion for we used retention of only two weeks, that, was not very strict. We suppose that, to evaluate robust bias mitigation interventions that are useful for practical purposes, more longitudinal bias reduction studies, lasting at least one year, are needed.

Regarding retention, there were a few positive findings for several biases. Effects were observed up to 12 weeks after the training interventions, with relatively large effect sizes (e.g., Clegg et al., 2015; Morewedge et al., 2015; Rhodes et al., 2017). The largest effect sizes were observed in studies that involved repeated game playing (Dunbar et al., 2014, 2017; Clegg et al., 2015). This agrees with the finding that repetitive training increases retention (Cepeda et al., 2008). Overall, serious games were by far the most successful interventions. Studies that specifically compared the effects of game-based with other kinds of interventions (eight in total) showed that games were more effective than video interventions (Veinott et al., 2013; Clegg et al., 2014, 2015; Dunbar et al., 2014, 2017; Morewedge et al., 2015; Rhodes et al., 2017; Shaw et al., 2018). The authors of these studies often suggest that the interactive and engaging nature of games might be the essential factor explaining this difference. The gaming studies yielded valuable information on retention of bias mitigation for a group of only six biases that are considered relevant for military intelligence analysis (Heuer, 1999; Morewedge et al., 2015). These were: Confirmation bias, Fundamental attribution error (Correspondence bias), Bias blind spot, Anchoring bias, Representativeness bias, Social projection. To be of value for other application domains, the interventions should also target other biases, such as: hyperbolic time discounting, sunk-cost fallacy, outcome bias, or statistical biases.

Studies on transfer of training beyond the intervention context were even more rare than those on retention. Our search yielded only one study in which the measurement of transfer (as well as retention) involved real behavior transcending the specific training conditions, problem structures, and domains (This was a study of Sellier et al., 2019), who observed a transfer effect of a game to a different problem and context in a field setting for about 19% of their student participants. This low number of subjects showing transfer of debiasing cannot be considered as a “substantial” transfer-of-training effect. This marginal evidence that is available for transfer beyond the training situation suggests that bias mitigation effects of training interventions may often be the outcome of a learned “trick,” a learned rule, or a manner to show desired behaviors in a specific task context, while the fundamental underlying decision making competences do not change. If the training interventions would indeed affect the actual underlying cause of biased thinking, behavioral transfer to other task domains and contexts should be more extensively demonstrable.

A few more things are worth noting about the studies discussed so far. The test-retest reliability for the anchoring bias was found to be low (Gertner et al., 2016). This could mean that individuals are more prone to anchoring in one context than in another. However, since the questionnaires that were used also had low correlations between their items (i.e., low internal consistency), it is not clear whether this bias is unstable over time or whether their assessment was flawed, for example by a too low number of relevant items in questionnaires (see e.g., Stanovich and West, 1998, 2001). Hence, no conclusions can be drawn about mitigation of the anchoring bias. Secondly, most studies involved only one training session, while repetition of training has been shown to increase retention effects in many different tasks and learning situations (e.g., Ausubel and Youssef, 1965; Cepeda et al., 2008). The present review found three out of the four studies with multiple training sessions indicating that playing the game twice resulted in more bias reduction than playing the game only once (Dunbar et al., 2014, 2017; Clegg et al., 2015). Lastly, there could be an effect of testing. Rhodes et al. (2017) investigated this and indeed found that testing alone reduced biases to some extent, but not as much as the interventions.

Given that this important subject of cognitive biases has been studied and documented extensively for a long time (Tversky and Kahneman, 1974), the limited number of relevant publications on retention and transfer of cognitive bias mitigation training is quite disappointing. Transfer has been studied substantially in specific limited areas in psychology like behavior modeling training (Taylor et al., 2005) and error management training (Keith and Frese, 2008). It is not *a priori* likely, however, that cognitive bias mitigation transfers to other areas, since it has been observed that transfer of training often fails (Burke and Hutchins, 2007). Another possibility is that retention and transfer of debiasing interventions have in fact been studied extensively, however without yielding significant results, except for serious gaming studies. Due to the phenomenon of “publication bias” (Dickersin, 1990) the “negative” non-gaming results may have resulted in these studies not being published. Also, debiasing interventions may have been reported using different terms than the ones used in the present literature search. There are studies aiming at an improvement in decision making in a broader sense that do not use the term “cognitive bias” (Fong and Nisbett, 1991; Kostopoulou et al., 2017). Indeed, we found some studies on retention of bias mitigation that did not include any of the expected keywords.

A minimal proof of retention and transfer of the training-effects of bias mitigation interventions may be grounded on theoretical insights. Biases (and heuristics) are typically explained with the Dual Processing Model (Stanovich and West, 2001; Kahneman, 2003, 2011a; Evans, 2008). This framework assumes that cognitive information processing can take place in two ways: the first way (“System 1,” heuristic) demands little effort and works quickly and automatically. This is the default way of thinking that feels self-evident and is sensitive to biases. The second way of thinking (deliberate, “System 2”) is slower, and demands effort and concentration. The “System” terminology was originally proposed by Stanovich and West (2001) and adopted by many scientists like Kahneman (2011a). It suggests distinct underlying brain systems that mediate emotional vs. rational reasoning (Feldman Barrett, 2017). This terminology is highly misleading and was merely adopted to make the dual processing model more easily comprehensible (Kahneman, 2011b), not to provide a fundamental explanation for cognitive biases. This means that the Two-System model should be considered mainly descriptive, instead of explanatory. It does not provide an explanation in terms of underlying mechanisms that cause cognitive biases that are robust over time and task conditions.

More explanatory power with regard to the origin of biases than the dual-processing framework is provided by a recent theoretical model for cognitive biases (Korteling et al., 2018; Korteling and Toet, 2020). According to this model biases are largely caused by structural (or ‘inherent) neural and ingrained evolutionary characteristics and mechanisms of the brain. Neural biases arise from the *inherent* characteristics of the functioning of the brain as a biological neural network, which was originally developed and optimized for biological and perceptual-motor functions. These characteristics distort or skew information, just like perceptual illusions (Reeves and Pinna, 2017). Basically, these mechanisms—such as association, facilitation, adaptation, or lateral inhibition—result in a modification of the original or available data and its processing (e.g., by weighting its importance). For example, lateral inhibition (Isaacson and Scanziani, 2011) is a universal neural process resulting in the magnification of differences in neural activity (contrast enhancement), which is very useful for perceptual-motor functions. However, for higher cortical functions, that require exact calculation and proper weighting of data and the application of the rules of logic and probability, this transformation of data may work out detrimentally. In these cases, this structural mechanism may lead to “contrast enhancement,” i.e., generating increasing differences in weighing up alternatives or selectively overweighing thoughts that occasionally pop up (cf. Availability bias: the tendency to judge the frequency, importance, or likelihood of an event by the ease with which relevant instances come to mind; Tversky and Kahneman, 1973, 1974). Because of such inherent, structural properties, our neural system will never be able to execute a command like “*Do not think of a pink elephant*” or to control which information pops up in our mind when deliberating about a (complex) issue. All stimuli entering the nervous system affect its structure and thereby its connectionist properties. So, unlike computer software, once information has entered the brain, it cannot simply be erased or made undone. Relating this example of neural information processing to biases: irrelevant information or counter-productive information (which has been provided) is always associatively integrated into the brain's physical-chemical structure. It is captured in the brain's neural circuitry and thus may (associatively) affect a following judgement or decision. This means that judgement and decision making is by definition always affected to some extent by persisting (“anchoring”) effects of information that has been processed before. Biased decision making may then occur when irrelevant or misleading information associatively interferes with the reasoning process. Examples of these neurally inherent biases are for instance Anchoring bias (biasing decisions toward previously acquired information or the “anchor”: Tversky and Kahneman, 1974; Furnham and Boo, 2011), and the Hindsight bias (the tendency to erroneously perceive events as inevitable or more likely once they have occurred: Hoffrage et al., 2000; Roese and Vohs, 2012).

In addition to the inherent (or structural) characteristics of (biological) neural networks, biases may also originate from ingrained evolutionary (or functional) heuristics that promoted the survival of our ancestors who lived as hunter-gatherers for hundreds of thousands of years in close-knit groups (Tooby and Cosmides, 2005; Haselton et al., 2009). Cognitive biases can be caused by mismatches between evolutionarily rationalized heuristics (“evolutionary rationality”: Haselton et al., 2009) and the modern environment or context in which we live (Tooby and Cosmides, 2005). From this perspective, the same thinking patterns that optimized the chances of survival of our ancestors in their (natural) environment can lead to maladaptive (biased) behavior when they are used in our current (artificial) settings. Biases that may be considered as examples of this kind of mismatch are: Authority bias (the tendency to attribute greater accuracy to the opinion of an authority figure -unrelated to its content- and to be more influenced by that opinion: Milgram, 1963, 1974), Conformity bias (the tendency to adjust one's thinking and behavior to that of a group standard: Cialdini and Goldstein, 2004), and the Ingroup bias (the tendency to favor one's own group above that of others: Taylor and Doria, 1981). The hypothesis that cognitive biases originate from (neurally) inherent and (evolutionary) ingrained brain mechanisms (Korteling et al., 2018; Korteling and Toet, 2020) may explain why it is so difficult to obtain long-lasting debiasing effects outside the context of the laboratory setting in which these effects were acquired. It explains why heuristic thinking (“System 1 thinking”) seems our default and intuitive way of thinking, demanding little attention and effort and feeling so natural and obvious (Kahneman, 2011b). And it explains why we are so blind to own biases and typically feel quite confident about our intuitive decisions and judgments, even when we are aware of our cognitive biases (Pronin et al., 2002; Risen, 2015; Scopelliti et al., 2015).

Bias mitigation interventions only have real value when they help people to make better decisions in practical situations in a long-lasting way. Based on the literature, we conclude that there is currently insufficient evidence for transfer and retention of bias mitigation interventions. So far, only a limited number of studies (largely from the IARPA SIRIUS research program (Bush, 2017) have reported positive results with regard to retention. In addition, the questionnaire-results of these studies may also be considered as an indication of transfer effects, i.e., “*near transfer.”* This means that with regard to real behavioral transfer of bias mitigation to concrete field conditions, i.e., far transfer, which is essential in such a diverse real-life area as decision making, there is barely anything known to date. As called for by Larrick (2004) and confirmed by Ludolph and Schulz (2018), more extensive studies on true transfer are required to investigate whether these interventions can beneficially aid decision making in real life. In addition, we advocate to more rigorously investigate the “hard-wired” psycho-biological origins of biases and (based on the results) how human decision making can be enduringly improved in a broad array of practical contexts.

As a first step, the supposed inherent, ingrained and subconscious character of biased thinking makes it unlikely that simple and straightforward methods like education classes or awareness courses with slide shows will appear very effective to ameliorate most biases. Mitigating biases will probably always remain a major challenge, especially when striving for real long-term effects in all kinds of different (daily life) situations and contexts. This counts especially for the biases that originate from the previously mentioned inherent or structural characteristics of biological neural networks. The reason for this is that these neural biases are inextricably linked to the structural system properties of biological neural networks, like the human brain. Because of these inherent properties it is impossible for the brain to correctly adhere to principles like: “*search without selection*,” “*ignore information X completely*” or “*weigh all the factors that you know fairly*.” In contrast, evolutionary biases are more “functional” and may be conceived as inborn preferences or inclinations that had survival-value in primordial times. Therefore, in contrast to neural biases, evolutionary biases may be considered less fundamental to the workings of biological neural networks. This means that it may be easier to learn to suppress evolutionary inherited tendencies that used to promote survival and reproduction of our ancestors. Examples of such evolutionary adaptive biases are: striving for immediate reward (Hyperbolic discounting), striving for admiration by peers (Social comparison), or herd tendencies like following or copying the opinions or behavior of the majority (Social proof) or biases following from our limited “higher” cognitive functions, like statistics, probability reasoning or calculation, which have been developed only very recently in evolution (e.g., Cosmides and Tooby, 1994; Henshilwood and Marean, 2003; Petraglia and Korisettar, 2003). The literature, so far, indicates that it seems most effective to use serious (video) games. These interventions encourage and motivate people to actively deal with their biases. In addition, game-based interventions (or maybe simulations) immerse people into a learning environment in which the effects of biases are more directly perceived and experienced. In the ancestral human world, there was always a tangible link between behavior and environment. In this world our brain has evolved to be affected by perceptual-motor experiences that we (directly) see, hear, or feel with our senses (e.g., Moravec, 1988; Cosmides and Tooby, 1994; van Vugt et al., 2014; Korteling et al., 2018). This direct experience in serious games (Experiental learning) is enhanced, or enriched, by the active and interactive nature of video games (e.g., Jiusto and DiBiasio, 2006; Korteling et al., 2013). The debiasing effects of serious games may therefore be explained by the evolutionary fact that people's thinking and decision making is most effectively influenced by direct, (inter) active experiences instead of by more (indirect) conceptual and abstract information and reasonings.

However, the notion that many biases arise from more or less hard-wired brain mechanisms, means that mitigating them will always be an “uphill-battle” requiring substantial motivation, effort and perseverance. Therefore, we suppose that the most promising way of dealing with biases may be to improve the *environment* or *context* in which people make decisions instead of trying to directly improve their thinking capacities. For example, one could stimulate or impose the use of certain very strict working methods or aids with which the ingrained tendency to biased thinking can be *prevented or circumvented*. Examples of these are checklists or premortems (e.g., countering optimism bias by imagining what could make a project go wrong; Klein, 2007). In addition, it is well-known that for the execution of specific cognitive tasks (logical, analytical, computational), modern digital intelligence may be more effective than biological intelligence (Moravec, 1988; Korteling et al., 2021). Therefore, we conjecture that ultimately the development of digital decision support systems (supposedly based on artificial intelligence) may appear the most effective way leading to improved human decision making.

## Data Availability Statement

The original contributions presented in the study are included in the article/Supplementary Material, further inquiries can be directed to the corresponding author/s.

## Author Contributions

JK conceived the original idea and supervised this study. JG searched the literature and wrote the initial draft paper. AT critically reviewed the draft manuscript. All authors were actively involved in the revisions of the original drafts and in writing the final version.

## Conflict of Interest

The authors declare that the research was conducted in the absence of any commercial or financial relationships that could be construed as a potential conflict of interest.

## Publisher's Note

All claims expressed in this article are solely those of the authors and do not necessarily represent those of their affiliated organizations, or those of the publisher, the editors and the reviewers. Any product that may be evaluated in this article, or claim that may be made by its manufacturer, is not guaranteed or endorsed by the publisher.
